# Interstitial Pregnancy in the Third Trimester with Severe Preeclampsia: A Case Report and Literature Review

**DOI:** 10.1155/2020/9408501

**Published:** 2020-05-12

**Authors:** Shiho Nagayama, Hironori Takahashi, Shohei Tozawa, Risa Narumi, Rie Usui, Akihide Ohkuchi, Shigeki Matsubara

**Affiliations:** Department of Obstetrics and Gynecology, Jichi Medical University, Japan

## Abstract

An interstitial pregnancy that continues beyond the second trimester is a rare phenomenon. We report a patient with an interstitial pregnancy undiagnosed until the third trimester. A multiparous woman was referred to us because of preeclampsia at 26 weeks of gestation. The placental position was the right fundus, and color Doppler ultrasound revealed myometrial thinning and subplacental hypervascularity, leading to a suspicion of placenta accreta spectrum (PAS). Emergency cesarean section was performed at 28^1/7^ weeks of gestation due to severe preeclampsia. The right tubal horn to the isthmus of the fallopian tube bulged with placental adhesion and a part of the tube had ruptured, with the omentum adhering to the ruptured part. Interstitial and tubal isthmic pregnancy with uterine rupture was diagnosed.

## 1. Introduction

An ectopic pregnancy can cause massive bleeding or uterine rupture. In particular types of ectopic pregnancies, such as cesarean scar ectopies, placenta accreta spectrum (PAS) can occur. To avoid these catastrophic events, ectopic pregnancies are ideally diagnosed and terminated in the first trimester.

Interstitial pregnancy accounts for 2.0-3.0% of ectopic pregnancies [1]. Here, we report a patient with interstitial pregnancy undiagnosed until the third trimester. The case was characterized by: (1) misdiagnosis of PAS, (2) occurrence of preeclampsia (PE), and (3) uterine rupture with the omentum adhering to the ruptured site, preventing the catastrophic clinical features associated with uterine rupture.

## 2. Case Report

**Figure 1:**
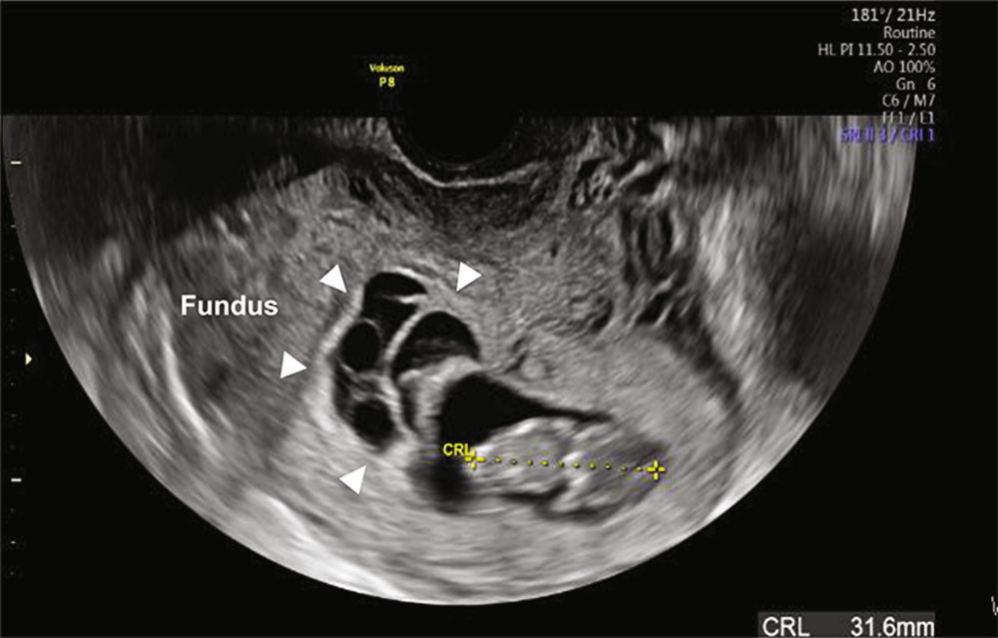
Transvaginal ultrasound showed subchorionic hematoma (arrowhead) located in the uterine fundus.

**Figure 2:**
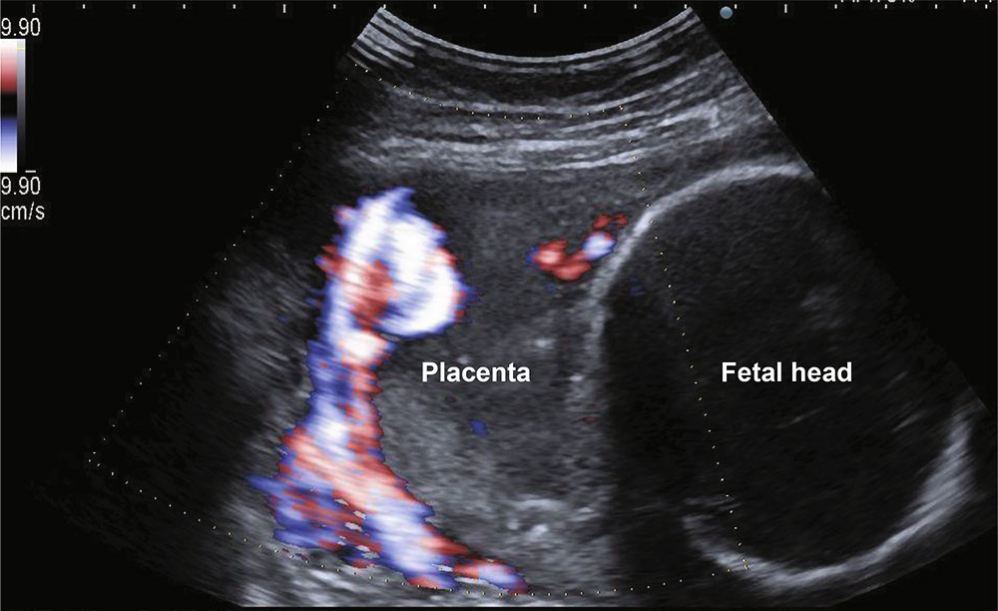
Color Doppler ultrasound revealed that the myometrium was unclear. The subplacental hypervascularity was noted at the back of the placenta.

A 41-year-old (4-gravida, 1-parous) Japanese women was referred to us because of early-onset PE (Eo-PE) at 26^6/7^ weeks of gestation. She had a history of two spontaneous abortions in the first trimester. She conceived naturally. Low-dose aspirin (LDA) (100 mg/day) was administered from 11 weeks of gestation because of recurrent abortion. She was diagnosed with subchorionic hematoma that was mainly located in the uterine fundus at 11 weeks of gestation without bleeding or pain (Figure 1). The hematoma disappeared at 16 weeks of gestation. The second trimester ultrasound showed no abnormal findings, and the course of pregnancy was uneventful until 24 weeks of gestation. At 26^6/7^ weeks of gestation, her blood pressure (BP) was found to be elevated (162/101 mmHg) with proteinuria (2.9 g/24 hours), and thus, she was admitted to this hospital. Nifedipine (20 mg/day) was started with BP at 140-160/80-90 mmHg. At 27^2/7^ weeks, proteinuria was 11 g/24 hours. Ultrasound revealed an estimated fetal weight of 940 g (-1.4 standard deviations) without growth arrest. The uterine artery and umbilical artery Dopplers were normal. A cardiotocogram also showed a reassuring pattern. The placental position was the right fundus and color Doppler showed subplacental hypervascularity at the back of the placenta (Figure 2), which led us to suspect PAS in the normal placental position (without previa).

**Figure 3:**
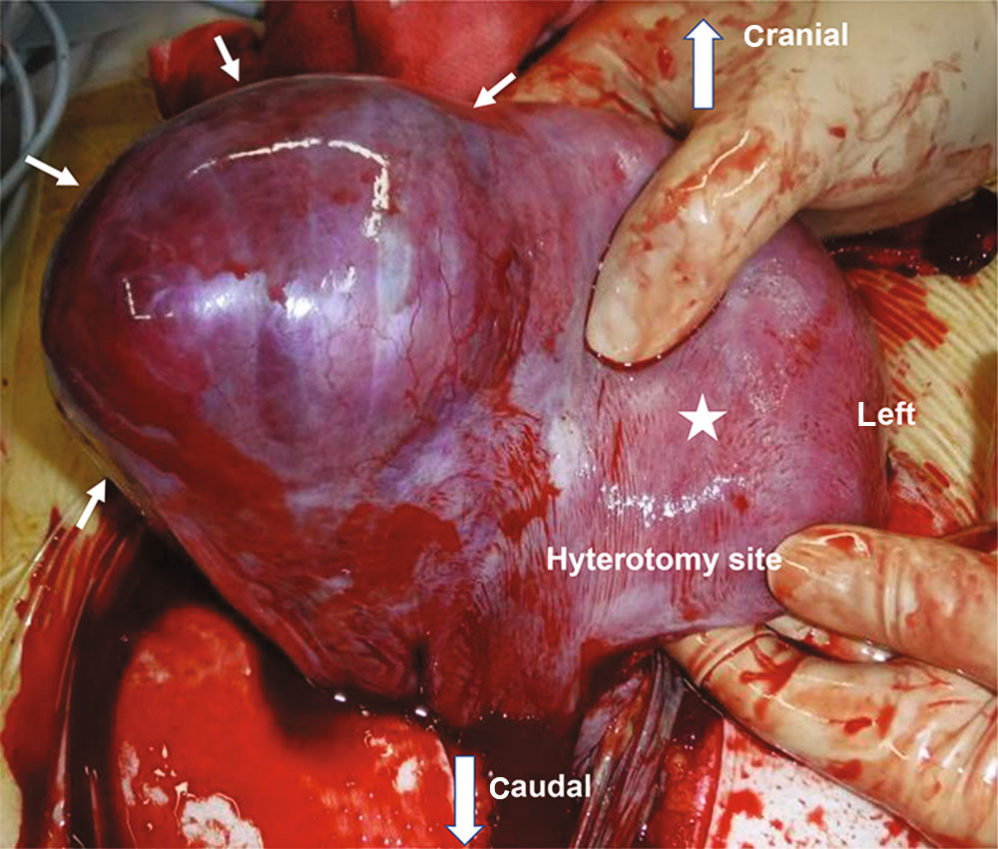
Intraoperative findings with a frontal view. The right tubal horn to isthmus of the fallopian tube bulged (arrow) with uterine rupture (reverse side of the uterus, not visible in this image). Uterus body is represented by a star.

**Figure 4:**
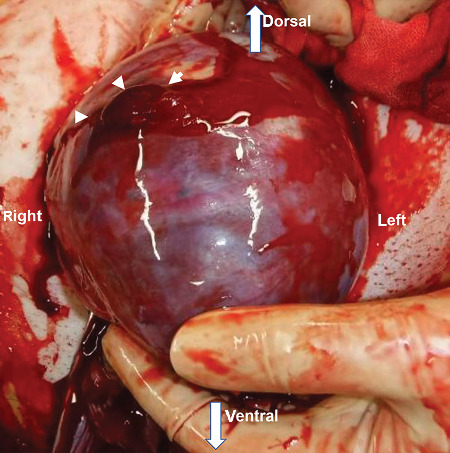
The part of the uterine rupture (arrowhead) masked by omental adhesion. This image was taken after removal of the omental adhesion.

**Figure 5:**
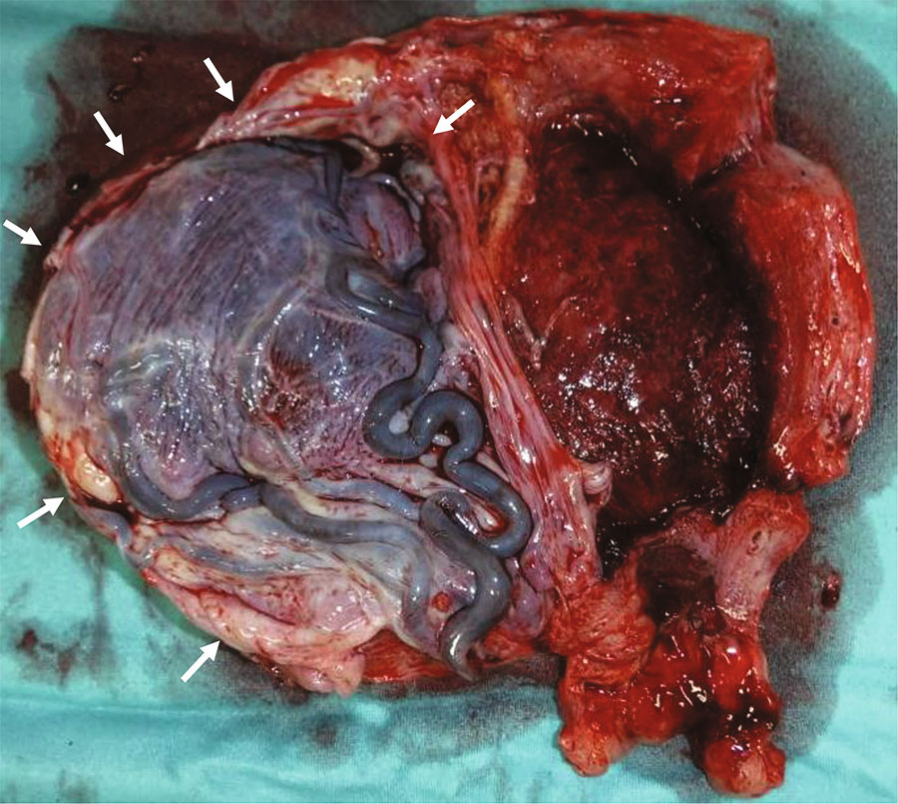
Macroscopic findings after hysterectomy. The placenta (arrow) was fully adherent to a thin interstitial part of the right fallopian tube.

She had severe edema of her legs and face. Chest X-ray also revealed lung edema; however, it was not severe and we administered betamethasone intramuscularly. Her blood pressure was over 180/110 mmHg under antihypertensives, and she also had severe headache. After a comprehensive analysis of all findings, an emergency cesarean section was conducted. The procedure was performed at 28^1/7^ weeks, yielding a female infant (926 g, Apgar score: 3/6 at 1/5 min, umbilical artery pH 7.44, B.E. -7.1 mmol/L). With the uterus exteriorized, the right tubal horn to isthmus of the fallopian tube showed bulging (Figure 3). A part of the fallopian tube (together with the adjacent uterus) had ruptured (Figure 4), with the omentum adhering over the rupture. Considering that preserving the uterus was impossible, we performed a hysterectomy. The intraoperative blood loss was 3,800 mL: 10 units of red blood cells and 8 units of fresh frozen plasma were transfused. The mother was discharged on the 10^th^ postoperative day with normal blood pressure (126/78 mmHg) without proteinuria or sequelae. The macroscopic findings revealed that the placenta had adhered to a thin interstitial and isthmic part on the right fallopian tube with uterine rupture (Figure 5). The pathological finding was interstitial pregnancy with placenta accreta. Interstitial and tubal isthmic pregnancy with uterine rupture was diagnosed. The infant was discharged at 3 months without sequelae.

## 3. Discussion

This is a case of interstitial pregnancy that continued until 28 weeks of gestation. This atypically long interstitial pregnancy gave us the chance to observe three important issues. This condition was misdiagnosed as PAS, PE, uterine rupture occurred with the ruptured site covered by the omentum, possibly preventing the catastrophic clinical features associated with the rupture; finally, the pregnancy was further complicated by Eo-PE.

To collect case reports about interstitial pregnancy with a live baby born in the third trimester, we searched for articles in PubMed and the Japan Medical Abstracts Society (https://login.jamas.or.jp) with the search terms of “interstitial pregnancy AND live” OR “interstitial pregnancy AND term” and found 273 articles (accessed on March 30, 2020, and published from 1991 to 2019). We read all abstracts, and there were 10 case reports that met the above criteria. Furthermore, we read references and related PubMed articles, resulting in 13 women including our case who were available for analysis (Table 1) [2–13].

The first important issue that this patient highlighted was interstitial pregnancy, if it is not diagnosed and terminated in early pregnancy, may show ultrasound features indistinguishable from, or at least mimicking, PAS. Indeed, in the present case, myometrial thinning and subplacental hypervascularity were evident, which are well-known ultrasound signs of PAS. These findings deceived us: we suspected the condition to be PAS based on these findings. In retrospect, we should have employed magnetic resonance imaging (MRI), which may have led to a diagnosis: no-PAS but pregnancy and its rupture or imminent rupture. However, whether MRI can be used to accurately diagnose this condition remains unknown with the literature detailing two cases of interstitial pregnancy diagnosed by MRI in the antepartum period. MRI is useful to diagnose interstitial pregnancy in the 1^st^ trimester [14]. D'Antonio et al. reported that MRI is also useful to diagnose PAS irrespective of whether patients have already undergone uterine surgery [15]. Given the usefulness of MRI in diagnosing interstitial pregnancy or PAS, MRI may be considered more actively.

Secondly, this case strongly suggests a cause-effect relationship between ectopic pregnancy and PE. This patient was multiparous, had no previous history of PE, and underwent LDA administration. LDA use for a high-risk PE patient decreases preterm PE by up to 63% [16]. The pathogenesis of Eo-PE comprises two processes, and the first process is inadequate trophoblast invasion [17]. To achieve adequate trophoblast invasion, the existence of sufficient decidua is essential. In normal pregnancy, endovascular and interstitial trophoblasts invade and remodel the spiral arteries in the decidua, facilitating maternal blood flow into the intervillous space. Hypoxia is maintained within the placental bed, as the early placenta is basically a hypoxic environment. However, the abnormal placental formation contributed to the persisting hypoxia, and, thus, trophoblast invasion and subsequent placental maturation were impaired. These are major risk factors for the development of Eo-PE. In this context, ectopic pregnancy may be a strong candidate for Eo-PE. In many cases of ectopic pregnancy, there is little or no trophoblast invasion due to the lack of or very thin decidua, becoming a similar condition to Eo-PE. Approximately 23% (3/13) of women with interstitial pregnancies were reported to develop PE (Table 1). When compared with the 3-5% of women who develop PE in a normal state, this suggests up to a 5-7-fold increased risk of PE. Although we cannot deny the possibility that PE was a coincidence, careful observation is needed regarding the relationship between ectopic pregnancy and PE.

The third finding was the fallopian tube and uterine rupture were hidden and catastrophic events associated with rupture may have been protected against by omental adhesion to the ruptured site. It is well-known that the omentum plays a defensive role against abdominal organ damage by its coverage. We previously reported patients in whom the omentum, adhering to the site of uterine rupture, prevented massive bleeding and catastrophic events and protrusion of the placenta, cord, and fetus into the abdominal cavity [18]. We referred to this condition as “masked rupture” [18, 19].

## 4. Conclusion

In conclusion, we described a patient with interstitial pregnancy, in whom pregnancy continued until the third trimester. This condition may mimic PAS. Although we suggest a possible association between intestinal pregnancy and PE, further study is necessary to confirm this.

## Figures and Tables

**Table 1:** Interstitial pregnancy in the third trimester.

Number	Author	Age	Antepartum MRI	HDP including preeclampsia	Rupture	Delivery weeks	Delivery	Operation	Fetus/ Neonate
1	Tanaka (2014) [2]	35	Not done	−	−	32	C/S for NRFS, breech presentation	C/S only^i^	Live
2	Okazak (2018) [3]	38	Not done	+	−	37	C/S for a history of C/S	Partial resection (Left tube and myometrium)	Live
3	Maeda (1991) [4]	26	Not done	+	−	36	C/S for placenta previa, horizontal presentation	Partial resection (Left tube, left ovary and myometrium)	Live
4	Ng (2007) [5]	25	Not done	−	−	38	C/S for breech presentation	C/S only^ii^	Live
5	Milićević (2010) [6]	36	Not done	−	−	Term	C/S for malpresentation	Hysterectomy^iii^	Live
6	Ugwumadu (1997) [7]	NA	Not done	−	+	33	C/S for NRFS	Hysterectomy^iii^	Live
7	Nishikawa (1998) [8]	31	Not done	−	−	37	C/S for breech presentation	Hysterectomy^iii^	Live
8	Scarella (2012) [9]	30	Myometrial infiltrating placenta, interstitinal pregnancy	−	−	28	C/S for NRFS and PPROM	Hysterectomy	Neonatal death
9	Bond (1988) [10]	34	Not done	−	−	39	C/S for breech presentation	Hysterectomy^iii^	Live
10	Rosenzweig (1998) [11]	29	Not done	−	−	38	C/S for abdominal pain	Hysterectomy^iii^	Live
11	Okada (2008) [12]	NA	Not done	−	+	34	C/S for uterine rupture	Hysterectomy^iii^	Live
12	Hill (2013) [13]	27	Interstitinal pregnancy	−	−	32	C/S for interstitinal pregnancy	Partial resection (right tube and right cornua)	Live
13	Our case (2019)	41	Not done	+	+	28	C/S for preeclampsia	Hysterectomy^iii^	Live

C/S, cesarean section; HDP, hypertensive disorders of pregnancy; HELLP, hemolysis, elevated liver enzyme, low platelet syndrome; MRI, magnetic resonance imaging; NA, not available; NRFS, non-reassuring fetal status; PPROM, preterm premature rupture of the membrane; ^i^The placenta was delivered naturally 8 days after delivery, ^ii^The placenta was delivered naturally 17 days after delivery, ^iii^supracervical hysterectomy.
